# The effects of infliximab therapy on the serum proteome of rheumatoid arthritis patients

**DOI:** 10.1186/ar2637

**Published:** 2009-03-06

**Authors:** Ravi C Dwivedi, Navjot Dhindsa, Oleg V Krokhin, John Cortens, John A Wilkins, Hani S El-Gabalawy

**Affiliations:** 1 Manitoba Centre for Proteomics and Systems Biology, University of Manitoba, 799-715 McDermot Avenue, Winnipeg, MB, R3E 3P4, Canada; 2 Department of Internal Medicine, University of Manitoba, RR149-800 Sherbrook Street, Winnipeg, MB, R3A 1M4, Canada

## Abstract

**Introduction:**

Although the clinical effects of infliximab therapy in rheumatoid arthritis have been documented extensively, the biological effects of this intervention continue to be defined. We sought to examine the impact of infliximab therapy on the serum proteome of rheumatoid arthritis patients by means of a mass spectrometry-based approach.

**Methods:**

Sera from 10 patients with rheumatoid arthritis were obtained prior to and following 12 weeks of infliximab therapy using a standard clinical protocol. The sera were immunodepleted of the 12 highest abundance proteins, labeled by the iTRAQ (isobaric tagging for relative and absolute protein quantification) technique, and analyzed by mass spectrometry to identify proteomic changes associated with treatment.

**Results:**

An average of 373 distinct proteins were identified per patient with greater than 95% confidence. In the 3 patients demonstrating the most robust clinical responses, changes of greater than 20% in the serum levels were observed in 39 proteins following treatment. The majority of these proteins were regulated directly or indirectly by tumour necrosis factor-alpha (TNF-α) and nuclear factor-kappa-B, with acute-phase proteins being uniformly down-regulated. A number of proteins, including members of the SERPIN family and S100A8, were down-regulated irrespective of clinical response.

**Conclusions:**

The present study demonstrates that a robust clinical response to infliximab is associated with the down-regulation of a spectrum of serum proteins regulated by TNF-α, and provides a possible basis for defining the broader biological effects of the treatment *in vivo*.

## Introduction

Rheumatoid arthritis (RA) is a clinically and biologically heterogeneous disorder. Response to disease-modifying anti-rheumatic drug (DMARD) therapy is unpredictable, even in patient groups that appear to be clinically homogeneous. The assessment of clinical response to DMARD therapy involves the acquisition and integration of patient-derived parameters measured by visual analogue scales and functional assessments such as the Health Assessment Questionnaire (HAQ), physician-derived counts of swollen and tender joints, and laboratory measures of the acute-phase response, usually the level of C-reactive protein (CRP) or erythrocyte sedimentation rate (ESR). For clinical trial purposes, these parameters are used to calculate a single composite index, with the disease activity score (DAS) being one of the most commonly used indices in RA clinical metrology [1,2]. Despite the widespread use of these indices in clinical trials, their performance as indicators of response in individual RA patients is more problematic [1]. Moreover, the biological basis for the clinical responses is not well understood. There is thus an important need for the development of biomarkers that more accurately reflect the impact of specific therapies on the underlying disease process.

With the introduction of targeted biological anti-rheumatic drug therapies, for which the mechanism of action is well defined, there has been an increased understanding of pathogenic mechanisms underlying RA [3]. In particular, the highly successful use of tumour necrosis factor-alpha (TNF-α) inhibitors in clinical practice has highlighted the central role that this cytokine plays in the pathogenesis of RA [4,5]. The biological effects of this treatment have been evaluated at both the systemic [6,7] and synovial [8-10] levels, although it remains unclear how each of these contributes to the overall clinical responses in the treated patients as measured using indices such as the DAS. Importantly, the biological mechanisms underlying a primary lack of response, a phenomenon observed in at least one third of treated RA patients, remain to be defined.

The availability of highly sensitive proteomic platforms provides the opportunity for a broad uncensored exploration of the changes that occur in the proteome with the use of targeted anti-rheumatic drug therapies such as infliximab, a potent TNF-α inhibitor now widely used in routine clinical practice. These approaches can be applied to a spectrum of biological materials, including serum, urine, synovial fluid, and cell populations. Because of their ease of acquisition in clinical practice, serum and plasma are well suited for assessing the effects of drug therapy in RA, but they are some of the most complex biological protein mixtures to analyze. In part, this relates to the preponderance of several high-abundance proteins such as albumin and gammaglobulin [11]. These high-abundance proteins mask the presence of potentially more informative low-abundance proteins, which are present at concentrations that are orders of magnitude lower. These multi-fold quantitative differences in the levels of serum proteins have necessitated the implementation of approaches that aim to reduce the complexity of the serum proteome by removing a spectrum of high-abundance proteins to unmask the lower abundance proteins, prior to undertaking the actual proteomic analyses [11].

We undertook a study evaluating the effects of infliximab on the serum proteome. We used a technique that depleted the 12 most abundant serum proteins, and then we labeled the proteins using the iTRAQ (isobaric tagging for relative and absolute protein quantification) technique to generate quantitative data. Thus, the proteome of a serum sample obtained at baseline was compared with that of a sample obtained after 12 weeks of infliximab therapy. The data generated support the utility of this approach in defining quantitative changes that occur in a wide spectrum of low-abundance proteins, and they demonstrate consistent changes in TNF-α-regulated proteins, particularly in the patients who had the most robust clinical responses.

## Materials and methods

### Patients

Ten patients who met American College of Rheumatology criteria for RA were included in this study [12]. The study protocol was approved by the Research Ethics Board of the University of Manitoba, and all patients provided informed consent. At the time of inclusion into the study, the patients were all receiving methotrexate and had demonstrated an incomplete response to optimum methotrexate doses of 15 to 20 mg weekly. A study rheumatologist undertook all clinical assessments. A baseline serum sample (T0) was drawn for proteomic analysis prior to initiation of infliximab. Patients were then treated with infliximab using a standard clinical protocol of 3 mg/kg at weeks 0, 2, and 6 and then every 8 weeks thereafter. A second serum sample was obtained for proteomic studies at week 12 (T12), at which time all patients had received three doses of infliximab. Patients were followed clinically for 52 weeks.

### Depletion of high-abundance serum proteins

Serum samples (30 μL) were processed to remove the 12 most abundant serum proteins (albumin, IgG, IgM, IgA, transferrin, fibrinogen, alpha2-macroglobulin, alpha1-anti-trypsin, haptoglobin, alpha1-acid glycoprotein, apolipoprotein A-I, and apolipoprotein A-II) using antibody-based immunodepletion spin columns in accordance with the protocol of the manufacturer (ProteomeLab™ IgY-12 high-capacity spin column; Beckman Coulter, Inc., Fullerton, CA, USA). In short, serum was incubated on an IgY-12 column for 20 minutes at room temperature and the depleted serum in the unbound flow-through was collected by centrifugation at 1,000 revolutions per minute for 30 seconds. The retained proteins were removed from the column and neutralized using buffers provided by the manufacturer. The regenerated column was used for the processing of subsequent samples.

### SDS-PAGE analysis

SDS-PAGE was performed to determine the quality of the sample preparations. Twenty microlitres of samples containing approximately 20 μg of serum, flow-through, or eluted IgY-12-bound proteins was denatured and reduced by boiling after combining with 5 μL of 5× SDS loading buffer containing 100 mM dithiothreitol (DTT) for 5 minutes. Twenty microlitres of each fraction was loaded and separated on 4% to 12% gradient SDS-polyacrylamide (Invitrogen Canada Inc., Burlington, ON, Canada). The gels were stained with gel-code blue (Pierce, Rockford, IL, USA).

### Sample preparation

Approximately 140 μg of processed serum proteins (as determined by the micro-bicinchoninic acid method; Pierce) obtained after IgY-12 column treatment was adjusted to a 200 μL volume with 100 mM ammonium bicarbonate buffer. The proteins in each sample were reduced with 10 mM (final concentration) DTT for 40 minutes at 56°C followed by alkylation using 50 mM iodoacetamide (IAA) for 20 minutes at room temperature. Excess of IAA was neutralized by the addition of 17 mM DTT for 20 minutes at room temperature. Proteins were digested in a 1:50 trypsin/protein ratio for 16 hours at 37°C. Samples were frozen at -20°C and dried using a speed vacuum. Trypsin-digested peptides were purified using a reversed-phase Scalar C-18 (1 × 100 mm, 5 μm, 100 Å) column (Agilent Technologies, Inc., Santa Clara, CA, USA).

### iTRAQ labeling of peptides

Aliquots of 140 μg of each isolate were digested separately with trypsin, and the resulting peptides were labeled with different reporter iTRAQ (Applied Biosystems, Foster City, CA, USA) in accordance with manufacturer procedure. Labeled samples were mixed in equal proportions and subjected to two-dimensional high-performance liquid chromatography (LC)-mass spectrometry (MS) analysis.

iTRAQ labeling allows for the simultaneous comparison of multiple samples in a single MS analysis [13,14]. This approach reduces some of the variability that can be encountered when comparing the results of samples that were analyzed in different experiments. The procedure involves the labeling of peptides from separate samples with an isobaric tag that contains one of the unique mass tags (for example, 114, 115, 116, or 117). The labeled peptides from the comparator samples are then combined in equal amounts and analyzed by MS. Thus, the basic premise of our analysis was that most proteins would be unchanged between T0 and T12 and would have reporter ion ratios of approximately 1 for their component peptides. Proteins that have alterations in their concentrations will deviate above or below this ratio, depending on the direction of the change.

### Two-dimensional high-performance liquid chromatography-mass spectrometry analysis

#### First dimension: peptide fractionation at pH 10

The peptides were separated using a recently developed two-dimensional LC method that employs high-pH reversed-phase separation in the first dimension [15-17]. Mixed iTRAQ-labeled peptides derived from approximately 120 μg of total protein were gradient-fractionated on a C18 X-Terra column (1 × 100 mm, 5 μm, 100 Å; Waters Corporation, Milford, MA, USA). Both eluents A (water) and B (90% acetonitrile) contained 20 mM ammonium formate buffer (pH 10.0). A total of 60 fractions were collected using a gradient of 1% to 50% of solvent B in 67 minutes at a flow rate of 150 μL/minute. Fractions were dried and dissolved in 50 μL of eluent A (see next section), and 20 μL was injected for analysis in the second dimension.

#### Second dimension: liquid chromatography-electrospray ionization-tandem mass spectrometry analysis

A splitless nano-flow Tempo LC system (Eksigent, Dublin, CA, USA) with sample injection via a PepMap100 trap column (0.3 × 5 mm, 5 μm, 100 Å; Dionex Corporation, Sunnyvale, CA, USA) and a 100 μm × 150 mm analytical column packed with 5 μm Luna C18(2) (Phenomenex, Torrance, CA, USA) was used in the second-dimension separation prior to tandem MS (MS/MS) analysis. Both eluents A (2% acetonitrile in water) and B (98% acetonitrile) contained 0.1% formic acid as an ion-pairing modifier. A 0.44% acetonitrile per minute linear gradient (0% to 35% B in 80 minutes, 500 nL/minute) was used for peptide elution, followed by a 5-minute wash with 80% B.

A QStar Elite QqTOF mass spectrometer (Applied Biosystems) was used in standard MS/MS data-dependent acquisition mode with a nano-electrospray ionization source. Survey MS spectra were collected (m/z 400 to 1,500) for 1 second followed by three MS/MS measurements on the most intense parent ions (80 counts/second threshold, +2 to +4 charge state, and m/z 100 to 1,500 mass range for MS/MS), using the manufacturer's 'smart exit' and 'iTRAQ' settings. Parent ions previously targeted were excluded from repetitive MS/MS acquisition for 60 seconds (mass tolerance of 50 mDa).

### Database search and protein identification

The MS/MS data were analyzed using ProteinPilot software version 2.0.1 (Applied Biosystems/MDS Sciex, Concord, ON, Canada). The search parameters were complete modifications of Cys alkylation with IAA, and inbuilt iTRAQ analysis residue modifications settings were on. Those protein candidates with greater than or equal to 95% identification confidence were used for further analysis.

### Functional categorization of proteins identified in serum

The annotation of protein cellular localization and biological function was performed using Ingenuity software (Ingenuity Systems, Inc., Redwood City, CA, USA).

## Results

All of the patients studied were females with a mean age of 45.5 years (range 33 to 72) and a mean disease duration of 2.3 years (range 0.5 to 20), and all but one patient were seropositive for IgM rheumatoid factor. Disease activity was assessed using the DAS28 (DAS using 28 joint counts)-CRP method [18]. The mean (range) of the baseline DAS28 for the patients was 5.8 (3.8 to 7.4) (Table 1). Response to infliximab therapy was assessed by comparing the T0 and T12 DAS28 scores, and the European League Against Rheumatism (EULAR) response criteria were applied [19,20]. A 'good' response is defined as Δ (T0 minus T12) DAS28 of greater than 1.2 from baseline *and *a current DAS28 of less than 3.2, whereas a 'moderate' response requires a Δ DAS28 of greater than 0.6 and less than 1.2 *and *a current DAS28 of greater than 3.2 and less than 5.1. None of the patients had achieved a 'good' response at T12 (Table 1), but patients 13, 16, 18, and 23 had all achieved a 'moderate' response on the basis of these criteria. Moreover, one of these individuals (10618) actually had an increase in the CRP level despite a reduction of 2.5 in the DAS28. We elected to exclude this last patient from the 'responder' analysis since the increase in CRP was likely to be an important confounder in the serum proteomic analysis. Thus, subjects 10613, 10616, and 10623 were considered the most homogeneous responders (Rs), and for the purposes of the proteomic analyses, the remaining subjects were considered to be non-responders (NRs).

**Table 1:** Patients and their classification according to responses based on disease activity score and C-reactive protein level after 12 weeks of infliximab treatment

Patient ID number	DAS28		Δ DAS	EULAR DAS28 response^a^	CRP mg/L		Δ CRP
					
	Baseline (T0)	Week 12 (T12)			Baseline (T0)	Week 12 (T12)	
10611	5.7	5.2	0.5	NR	4.3	29.1	-24.8
10612	6.2	7.6	-1.4	NR	37.4	139.0	-101.6
10613	5.8	3.6	2.3	R	13.0	9.0	4.0
10616	6.8	3.9	2.9	R	46.0	1.0	45.0
10618	6.3	3.8	2.5	NR^b^	43.3	52.0	-8.7
10619	3.2	3.7	-0.5	NR	6.0	8.0	-2.0
10620	4.9	5.1	-0.2	NR	17.1	2.3	14.8
10621	3.8	4.2	-0.5	NR	68.0	68.0	0.0
10622	6.0	6.6	-0.6	NR	24.4	86.2	-61.8
10623	6.2	4.5	1.7	R	51.3	12.4	38.9

^a^Patients were deemed to be European League Against Rheumatism (EULAR) responders (Rs) if they had a reduction in DAS28 of greater than or equal to 1.2; all others were considered non-responders (NRs) for analysis purposes. ^b^Subject 10618 achieved a reduction in DAS28 of 2.5, but C-reactive protein (CRP) level increased at T12; thus, to ensure homogeneity in the R group, we excluded this patient from the group. DAS, disease activity score; DAS28, disease activity score using 28 joint counts.

**Figure 1:**
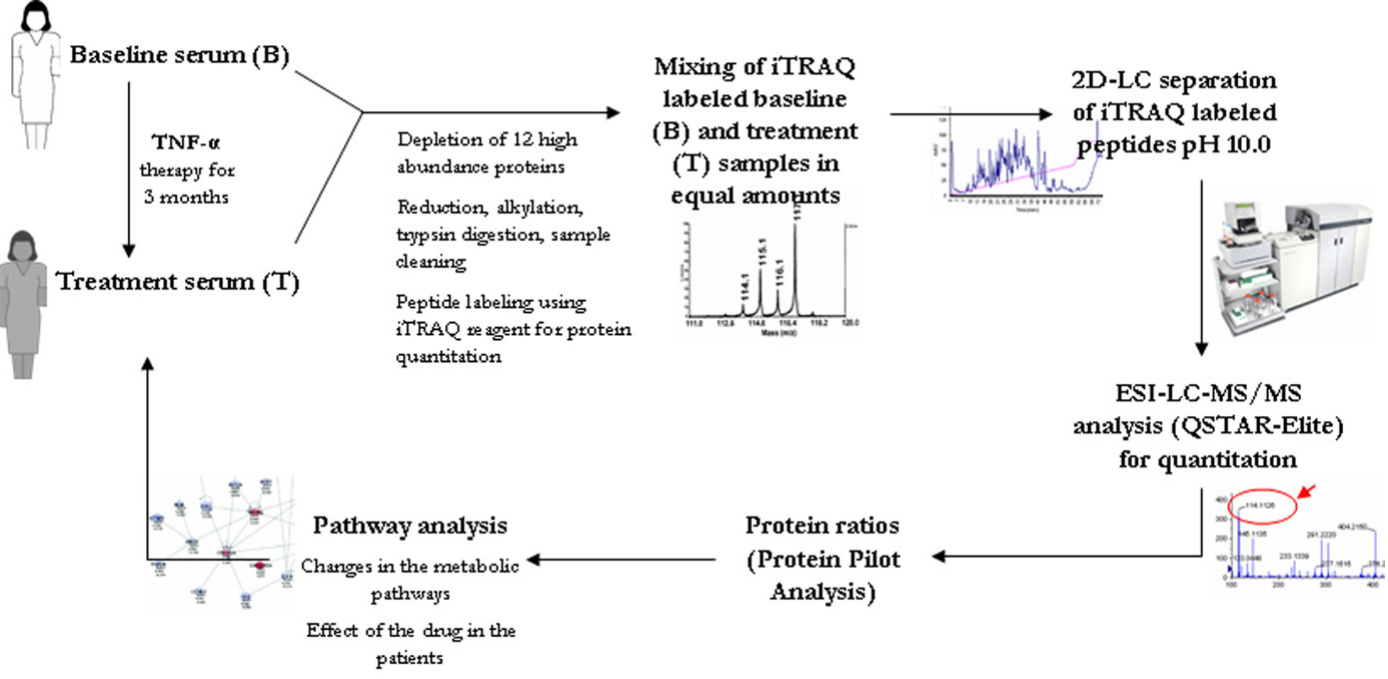
Experimental design of the study. 2D LC, two-dimensional liquid chromatography; ESI-LC-MS/MS, electrospray ionization liquid chromatography tandem mass spectrometry; iTRAQ, isobaric tagging for relative and absolute protein quantification; TNF-α, tumour necrosis factor-alpha.

The aim of this study was to compare the protein composition of the sera of RA patients at baseline (T0) and following 12 weeks of infliximab treatment (T12) to determine how protein composition changed during this period. The sera were immunodepleted of the 12 most abundant proteins as these proteins constitute more than 90% of the serum protein content by mass and their presence interferes with the identification of potentially more informative lower abundance species [11,21]. Protein estimations indicated that there were reductions of between 87% and 92% in the serum samples following IgY-based immunodepletion (Additional data file 1). This was further supported by a comparison of the SDS-PAGE analysis of starting serum, IgY flow-through, and the retentate from the same serum (see Additional data file 2 for representative examples). Nevertheless, based on their subsequent identification by MS, these high-abundance proteins were clearly not removed completely from the sera. The depleted serum samples were subsequently processed and labeled with iTRAQ reagent as shown in the work flow outlined in Figure 1.

The MS-MS analysis identified an average total of 697 proteins, of which 373 were identified with greater than or equal to 95% confidence (Additional data file 3). Only the latter proteins were used for quantitative comparisons. In total, 83 proteins were identified in all samples, whereas 108 were identified in 9 out of 10 samples. Importantly, within a given patient set, there was almost complete correlation between the proteins identified in the two serum samples. These data indicate that the inter-individual differences in the serum proteome are considerably larger than the intra-individual differences.

In analyzing the paired samples, we assumed that the majority of proteins would not substantially change in their relative levels and that, as such, the ratio for most proteins would remain at 1.0. Indeed, our analysis of these ratios indicated that nearly 80% of the proteins identified had a ratio of approximately 1.0, suggesting that the majority of the identified proteins were not significantly changed during treatment with infliximab. The remaining 20% of proteins displayed some variability between the T0 and T12 samples.

### Analysis of proteome changes based on clinical response

As indicated above, the clinical responses of the patients to infliximab were heterogeneous. Based on the data shown in Table 1 and the response criteria defined by Fransen and Van Riel [20], the patients were classified as optimum Rs or NRs, keeping in mind the limitations of the dichotomous classification discussed above. Thus, for the purpose of this analysis, patients 10613, 10616, and 10623 were classified in the R group, with the remainder classified in the NR group. On this basis, the proteins that changed significantly in either or both groups are shown in Table 2. There was a downregulation in the majority of these detected proteins, and where detected in both groups, this was quantitatively larger in the R group (Additional data file 4). Of note, the mean T12/T0 CRP ratio was 1.39 in the NR group, which was consistent with a mean increase in the CRP level of 26.3 mg/L as detected by nephelometry. A CRP ratio could not be accurately determined in the R group analysis as this protein was not detected in the T12 sample from 2 out of 3 patients with 95% confidence score, *P*-value of 0.001–0.05 and EF ≤ 2.0. The T12/T0 ratio for other proteins known to be associated with the acute-phase response, such as ceruloplasmin and complement proteins, was consistently lower in the R group compared with the NR group, suggesting that with successful therapy there was a greater reduction in the absolute levels compared with baseline.

**Table 2:** Proteins with changes between week 12 and baseline identified in the responder and non-responder groups

Protein name	Average ratio		Protein ID
		
	Responder	Non-responder	
Anti-thrombin III variant	1.27	0.86	gi|576554
Ceruloplasmin	0.80	0.95	gi|1620909
Complement component 3	0.84	0.92	gi|40786791
Complement component 5	0.89	0.94	gi|38016947
Complement component 7 precursor	0.65	0.89	gi|45580688
Complement factor H	0.82	1.06	gi|56203410
Keratin 1	1.72	0.69	gi|17318569
Keratin 2	1.60	0.70	gi|47132620
ORM2	0.52	0.64	gi|48145977
Orosomucoid 1	0.48	0.60	gi|55958974
Plasminogen	0.85	0.91	gi|56203917
Serum albumin precursor	0.72	1.18	gi|6013427
Unnamed protein product	1.10	0.86	gi|29581
Unnamed protein product	0.71	0.89	gi|1335098
Angiotensinogen (ser [or cys]) proteinase inhibitor	1.19	ND	gi|37790798
Anti-(ED-B) scFV	1.19	ND	gi|3152364
Apolipoprotein A1	0.69	ND	gi|4960066
C1 inhibitor	0.74	ND	gi|29535
CarboNDypeptidase N polypeptide 1 50 kD	0.73	ND	gi|55960072
Carnosinase 1	0.8	ND	gi|21071039
Coagulation factor NDII-Mie	1.11	ND	gi|24899162
Complement C1s	0.89	ND	gi|6407558
Complement component 1, r subcomponent	0.82	ND	gi|23243256
Complement component 2	0.87	ND	gi|55961814
Cystatin C	1.51	ND	gi|296643
Hypothetical protein	1.17	ND	gi|51476334
Insulin-like growth factor-binding protein	0.81	ND	gi|19344010
Inter-alpha-trypsin inhibitor heavy-chain H1	0.89	ND	gi|825630
Keratin 10	1.91	ND	gi|40354192
Leucine-rich alpha-2-glycoprotein 1	0.68	ND	gi|47125362
PeroNDiredoNDin 2 isoform b	0.52	ND	gi|33188452
Serpin peptidase inhibitor	0.83	ND	gi|50659080
Serpin peptidase inhibitor, clade F	0.79	ND	gi|21594846
SERPINC1 protein	0.75	ND	gi|18490839
Transferrin	0.72	ND	gi|37747855
Transthyretin	1.44	ND	gi|48145933
Unnamed protein product	0.87	ND	gi|29888
Vitamin D-binding protein precursor	0.88	ND	gi|139641
Vitronectin	0.86	ND	gi|14326449
Alpha1-anti-chymotrypsin	ND	1.06	gi|1340142
Alpha-2-glycoprotein 1, zinc	ND	0.82	gi|4502337
Apo-B100 precursor	ND	1.5	gi|28780
Apolipoprotein H precursor	ND	0.9	gi|4557327
Coagulation factor II precursor	ND	0.91	gi|4503635
Complement component C8 beta chain precursor	ND	0.91	gi|20141201
C-reactive protein	ND	1.39	gi|30224
Hp2-alpha	ND	0.47	gi|296653
IGHM protein	ND	1.37	gi|49256421
Immunoglobulin kappa L chain VLJ region	ND	1.38	gi|21669449
Mutant beta-globin	ND	1.29	gi|18418633
Protein S alpha	ND	0.88	gi|190442
SERPIND1	ND	0.84	gi|47678677
Unnamed protein product	ND	0.88	gi|28375497

All proteins had a greater than or equal to 95% confidence score for identification, *P*-value of less than or equal to 0.001 to 0.05, and an EF value of less than or equal to 2.0 and were present in two thirds of the patient group. ND, not determined. EF, error factor.

To understand the potential biological relevance of the changes in protein expression in this group, we examined the possible interactions of those proteins which were differentially regulated following the treatment. Out of the complete list of protein ratios identified in the R group (Additional data file 5) and the NR group (Additional data file 6), only those proteins that had a greater than or equal to 95% confidence score, *P*-value of less than or equal to 0.001 to 0.05, and an effort factor (EF) value of less than or equal to 2.0 and that were present in at least two thirds (≥66%) of this patient population were selected for pathway analysis. The output of these analyses indicates known direct or indirect interactions based on current literature and also serves to provide information about pathological states in which these have been observed.

**Figure 2:**
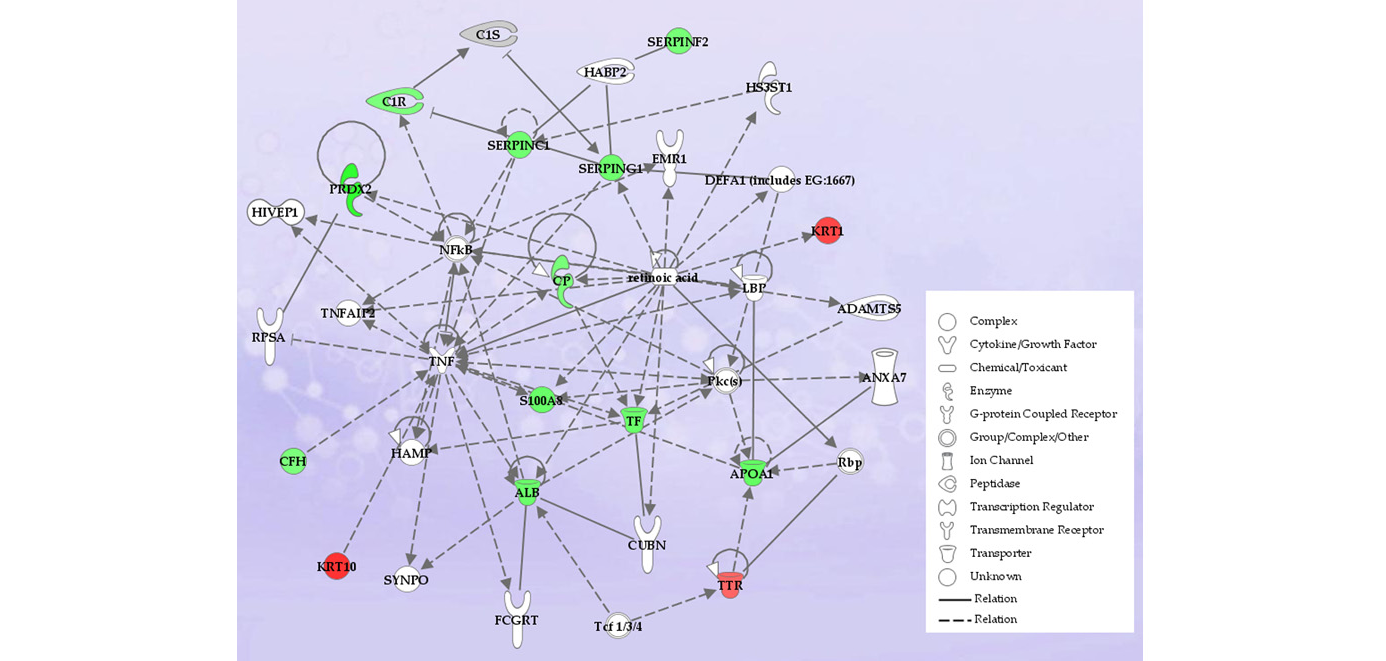
Ingenuity pathway analysis of proteins displaying alterations in expression patterns following infliximab treatment in patients deemed to be responders. (See Results for response definitions.) The network diagram shows the relationship between the indicated proteins by solid or dashed lines, which represent direct or indirect interactions, respectively. Up-regulated proteins are shown in red, and down-regulated proteins are shown in green. The network is centred on tumour necrosis factor-alpha (TNF-α) and nuclear factor-kappa-B (NF-κB) as reflected by the number of interactions associated with these proteins.

**Figure 3:**
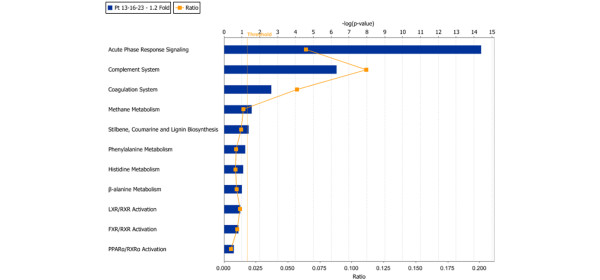
Ingenuity pathway analysis showing canonical pathways of the 39 differentially expressed proteins after infliximab treatment in the responder group. The pathways are indicated on the y-axis. The x-axis indicates the significance score (negative log of *P*-value calculated using Fisher exact test). FXR, farnesoid × Receptor; LXR, liver × receptor; PPARα, peroxisome proliferator activated receptor-alpha; Pt, patient; RXR, retinoid × receptor.

The 39 proteins identified in the R group, as shown in Table 2, were included in the pathway analysis (proteins with ≥ 1.2 fold ratio changes were used for network generation). This analysis used 23 functions/pathway-eligible molecules and 22 network generation-eligible molecules. As shown in Figure 2, the associated network featured TNF-α and nuclear factor-kappa-B (NF-κB) at the centre. The closest interacting partners of TNF-α and NF-κB (either direct or indirect interaction shown by solid or dashed arrows, respectively) were down-regulated. Moreover, the analysis indicated that the 'acute-phase response signaling' canonical pathway was most significantly affected in the R group, followed by the complement and coagulation pathways, as shown in Figure 3. As noted above, several of the complement proteins are acute-phase reactants and can be categorized in the former pathway.

**Figure 4:**
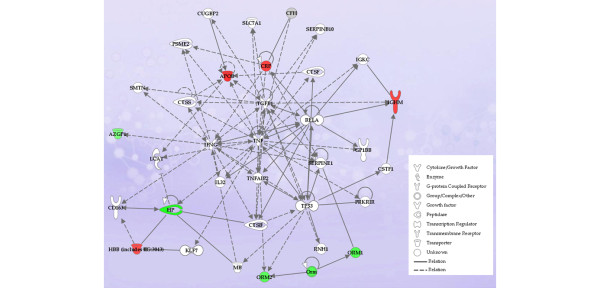
Ingenuity pathway analysis of proteins displaying alterations in expression patterns following infliximab treatment in patients deemed to be non-responders. (See Results for response definitions.) A network analysis of the differentially expressed proteins in the non-responder group indicates that, as with the responder network shown in Figure 2, tumour necrosis factor (TNF) is at the centre of the network but the down-regulated proteins are more distal, whereas more proximal proteins such as C-reactive protein (CRP) are up-regulated.

The 28 proteins identified in the NR group, as shown in Table 2, were similarly analyzed. These data are shown in Figure 4. It should be noted that, although this network also featured TNF-α prominently at the centre, the down-regulated molecules were more distally associated, whereas the proximal proteins such as CRP and apolipoprotein-A1 were up-regulated in this group. Thus, the profile of the differentially regulated proteins in the NR group was distinct from that of the R group. However, there were 2.7-fold fewer proteins that met the threshold criteria for inclusion in the NR group than in the R group. This may have excluded a number of interesting candidate proteins that could serve to distinguish infliximab responders from non-responders.

## Discussion

The present study examined the effects of a standard clinical infliximab therapy protocol on the serum proteome of RA patients. Since the biological target of this therapeutic monoclonal antibody is clearly defined to be TNF-α, we anticipated that this analysis might potentially provide mechanistic data on the systemic effects of this intervention. The results demonstrate the feasibility of this broad-based discovery approach, but they also point out the inherent difficulties in reconciling the clinical and biological effects of the treatment.

Inhibition of TNF-α has emerged as a highly effective therapy in many RA patients who have not responded to other forms of disease-modifying therapy such as methotrexate [4,5]. Yet the clinical response to TNF-α inhibitors is heterogeneous, as illustrated in the present study. Although the biological basis for this heterogeneity remains largely unknown, it can be speculated that the clinical response encompasses systemic effects as well as effects on the target tissue, the synovium. It is commonly observed that infliximab infusion has dramatic and rapid-onset effects on systemic features of RA such as fatigue, even after a single intravenous infusion. In contrast, the synovial effects of infliximab may not become apparent for several weeks, possibly several months. The primary time point chosen for analysis in this study, 12 weeks, is the point at which the majority of patients have a clinical response. It should be added that analysis of the clinical responses at subsequent time points did not reveal major changes in the trends observed at 12 weeks (data not shown).

Although numerous studies have attempted to define specific biomarkers for disease activity and response to therapy in RA, a limited number of proteomic studies have attempted to mine the entire proteome of the serum or synovial fluid for candidate biomarkers. One study used surface-enhanced laser desorption ionization (SELDI) to identify potential candidates and demonstrated that an increase in myeloid-related protein 8, possibly in a citrullinated form, was associated with RA, albeit non-specifically [22]. Liao and colleagues [23] used a two-dimensional LC MS/MS-based approach to analyze synovial fluid proteins with molecular weights of less than 40 kDa from patients with erosive or non-erosive RA (n = 5 per group). The relative abundance of proteins was determined using spectral counting. Subsequently, multiple reaction monitoring was used to examine the sera of another 15 donors (normal, erosive, and non-erosive RA) for the presence of the candidate biomarkers identified in the original studies. There was an increase in CRP and several S100 protein family calcium-binding proteins in the synovial fluids of patients with erosive RA compared with those with non-erosive disease. It was also observed that CRP, S100A8 (calgranulin A), S100A9 (calgranulin B), and S100A12 (calgranulin C) were markedly elevated in the sera of patients with erosive disease compared with the other groups.

Recently, a report comparing the proteome changes in sera of RA patients immediately prior to and 24 hours after infusion with infliximab was published [24]. Expression levels were compared for proteins with molecular weights of less than 30 kDa. The relative quantitation was based on the number of significant scoring peptides identified per protein, with increased numbers of peptides being interpreted as an indication of increased protein concentration. That study contrasts with ours in that we used a depletion method that selectively removed the most abundant serum proteins, thus allowing us to examine the full range of proteins. Moreover, we used a quantitation method, iTRAQ, which directly compares relative protein abundance rather than indirect comparative methods such as peptide or spectral intensity counts. Arguably, the most important difference between the two studies was the timing of the second sample, with ours being at 12 weeks, a time at which the clinical response to infliximab is evident.

Assessing the clinical response in individual RA patients is challenging. In part, this is a result of the heterogeneity of clinical states that are seen in RA and that are attributed to disease activity. Thus, an RA patient may feel subjectively better with treatment and have fewer tender joints and yet continue to have many swollen joints and elevated acute-phase reactants, whereas another RA patient may experience exactly the opposite. The two patients may demonstrate very similar changes in composite indices such as the DAS28. The clinical data generated in the context of the present study serve to illustrate these difficulties. In attempting to define a dichotomous R/NR outcome after 12 weeks of infliximab therapy, we used one of the best-validated approaches to data analysis, the EULAR response criteria [19]. Four individuals had sizable reductions in their DAS28 scores (mean Δ DAS28 of 2.35), although none achieved a 'good' EULAR response as they did not achieve a DAS28 of less than 3.2 at the 12-week time point. However, this pool of 4 Rs included an individual who, despite a reduction of 2.5 in DAS28, actually had a CRP increase at T12. We elected to exclude this individual from the R group in order to achieve the greatest degree of biological homogeneity in this group.

Since the present study focused on analyzing changes in the serum proteome in response to infliximab therapy, correlation with CRP level was particularly relevant to the analysis. CRP is the most sensitive biomarker for systemic inflammation and is widely used in clinical practice to guide RA therapy. In non-inflammatory conditions, this protein is virtually undetectable in the serum, and under the influence of pro-inflammatory cytokines, particularly interleukin-6, CRP synthesis and secretion by the liver increase several fold, along with a spectrum of other proteins collectively classified as acute-phase reactants [25]. This includes amyloid A protein, ceruloplasmin, haptoglobin, and several complement proteins. In the optimum-response R group, CRP was generally undetectable in the T12 samples and thus a ratio could not be derived. In the paired serum samples from the NRs, the mean CRP ratio detected by MS was 1.39, indicating an increase in CRP level, which correlated well with the levels as determined by nephelometry. This suggests that the proteomic ratios were indeed reflective of the actual protein levels in the serum. In the case of ceruloplasmin and several complement proteins known to be part of the acute-phase response, the ratios were consistently lower in the R group than the NR group, further supporting this conclusion.

In contrast to these effects on acute-phase proteins, which overall tended to follow expected patterns, a number of detected proteins were down-regulated in both Rs and NRs. These included SERPINC1, S100A8, PRDX2, C1R, APOA1, SERPINF2, SERPING1, ORM1, and ORM2 (Table 2 and Figures 2 and 4). Of note, the changes in S100A8 were identified by Sekigawa and colleagues [24] in their study of the short-term effects of infliximab and were also reported by Liao and colleagues [23] in their work to identify protein biomarkers for RA. The SERPINs are a family of serine proteases involved in a spectrum of biological pathways and are of particular relevance to the coagulation pathway. Hereditary deficiencies in SERPINC1 (anti-thrombin 3) are known to increase the risk of thrombosis [26]. The overall impact of infliximab therapy on the coagulation pathway is not well defined, but the available data suggest that key pro-coagulant proteins are reduced [27,28]. This is potentially of considerable importance since RA is known to increase the risk of thrombotic events such as myocardial infarction and this risk may be modified by TNF-α inhibitors [29]. The proteomic data generated from this study do not provide a clear indication of how the coagulation pathway is impacted, particularly since serum rather than plasma was analyzed. Nevertheless, the results do point to the fact that TNF-α inhibition may impact on coagulation proteins, potentially independently of its effects on inflammatory pathways. The ultimate impact on the risk of thrombotic events requires large longitudinal clinical studies.

In summary, we provide evidence that MS-based proteomic techniques using a labeling method such as iTRAQ can be used to generate quantitative data about the changes that occur in the serum proteome in the context of targeted therapeutic interventions such as infliximab. Since serum is one of the most complex biological fluids, depletion of high-abundance proteins such as albumin and gammaglobulin is a key step in allowing the detection of low-abundance but potentially informative proteins. Our study also points out the inadequacy of the currently available clinical methods for assessing disease activity in individual patients, particularly if the biological basis of these responses is to be understood.

## Conclusion

The present study demonstrates that a robust clinical response to infliximab is associated with the downregulation of a spectrum of serum proteins regulated by TNF-α, and provides a possible basis for defining the broader biological effects of the treatment *in vivo*.

## Abbreviations

CRP: C-reactive protein; DAS: disease activity score; DAS28: disease activity score using 28 joint counts; DMARD: disease-modifying anti-rheumatic drug; DTT: dithiothreitol; EULAR: European League Against Rheumatism; IAA: iodoacetamide; iTRAQ: isobaric tagging for relative and absolute protein quantification; LC: liquid chromatography; MS: mass spectrometry; MS/MS: tandem mass spectrometry; NF-κB: nuclear factor-kappa-B; NR: non-responder; R: responder; RA: rheumatoid arthritis; T0: time point at baseline; T12: time point at 12 weeks; TNF-α: tumour necrosis factor-alpha.

## Competing interests

This work was funded, in part, by an unrestricted research grant from Schering-Plough Canada (Kirkland, QC, Canada), and HSE-G has served as a consultant on scientific advisory boards for Schering-Plough Canada. The other authors declare that they have no competing interests.

## Authors' contributions

RCD designed the experiments, processed samples, acquired and analyzed the data, and participated in writing the manuscript. ND acquired the samples and the clinical data. OVK performed the mass spectrometry and biochemical separations. JC performed the bioinformatics analysis. JAW designed the study, analyzed the data, and participated in writing the manuscript. HSE-G designed the study, acquired clinical data, analyzed the study results, and participated in writing the manuscript. All authors read and approved the final manuscript.

## Supplementary Material

Additional file 1 Depletion efficiency and total amount of proteins recovered (μg) from 30 μl of serum in flow through after IgY-12 column treatment. The recovered proteins were further used for proteomic analysis. Click here for file

Additional file 2 SDS-PAGE analysis of proteins from serum and at different stages of IgY-12 depletion. (Lane (1) Marker, (2,5) Serum, (3,6) Flow through collected after depletion of proteins from IgY-12 column, and (4,7) proteins eluted from the column. Lane 2,3,4 represent T0 and lane 5,6,7 represent T12 stage of serum sample). Click here for file

Additional file 3 Summary of the Mass Spectrometry results for all 10 samples. Click here for file

Additional file 4 Summary of the numbers of proteins observed displaying alterations in expression levels following treatment (# of proteins *P *≤ 0.001–0.05, EF ≤ 2.0, ≥ 95% confidence for identification). Click here for file

Additional file 5 Proteins present in any of the R group that displayed ≥2 fold changes in relative expression levels. (*P *≤ 0.001–0.05, EF ≤ 2.0, ≥ 95% confidence for identification). Click here for file

Additional file 6 Proteins present in any of the NR group that displayed ≥ 2 fold changes in relative expression levels. (*P *≤ 0.001–0.05, EF ≤ 2.0, ≥ 95% confidence for identification). Click here for file
