# Supplementary material for: The effects of infliximab therapy on the serum proteome of rheumatoid arthritis patients
Source: Arthritis Res Ther. 2009 Mar 6;11(2):R32. doi: 10.1186/ar2637 (PMC2688177; doi:10.1186/ar2637)
Supplement: Additional file 1 — Depletion efficiency and total amount of proteins recovered (μg) from 30 μl of serum in flow through after IgY-12 column treatment. The recovered proteins were further used for proteomic analysis. [file ar2637-S1.doc]

**Additional File #1.** Depletion efficiency and total amount of proteins recovered (µg) from 30 µl of serum in flow through after IgY-12 column treatment. The recovered proteins were further used for proteomic analysis.

| **Patient ID #** | **% depletion** | **Protein conc. (µg)** | **% depletion** | **Protein conc. (µg)** |
| --- | --- | --- | --- | --- |
|  | Base line (B) | Base line (B) | Treatment (T) | Treatment (T) |
|  |  |  |  |  |
| 10611 | 87.3% | 312.6 | 92.3% | 182.9 |
| 10612 | 93.3% | 218.6 | 93.7% | 188.2 |
| 10613 | 89.9% | 197.3 | 93.4% | 144.9 |
| 10616 | 94.0% | 209.4 | 95.9% | 135.1 |
| 10618 | 90.6% | 183.5 | 88.9% | 232.5 |
| 10619 | 86.7% | 263.5 | 88.3% | 257.2 |
| 10620 | 91.0% | 201.1 | 90.8% | 208.2 |
| 10621 | 87.9% | 326.5 | 88.9% | 282.4 |
| 10622 | 91.2% | 219.4 | 87.8% | 284.6 |
| 10623 | 86.4% | 349.1 | 85.2% | 381.4 |
|  |  |  |  |  |
