# Supplementary figures and images for: The effects of infliximab therapy on the serum proteome of rheumatoid arthritis patients
Source: Arthritis Res Ther. 2009 Mar 6;11(2):R32. doi: 10.1186/ar2637 (PMC2688177; doi:10.1186/ar2637)

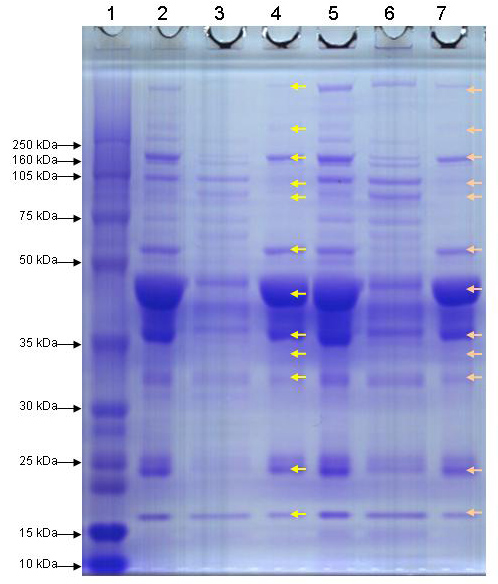

Supplement: Additional file 2 — SDS-PAGE analysis of proteins from serum and at different stages of IgY-12 depletion. (Lane (1) Marker, (2,5) Serum, (3,6) Flow through collected after depletion of proteins from IgY-12 column, and (4,7) proteins eluted from the column. Lane 2,3,4 represent T0 and lane 5,6,7 represent T12 stage of serum sample). [file ar2637-S2.tiff]
