# Supplementary material for: The effects of infliximab therapy on the serum proteome of rheumatoid arthritis patients
Source: Arthritis Res Ther. 2009 Mar 6;11(2):R32. doi: 10.1186/ar2637 (PMC2688177; doi:10.1186/ar2637)
Supplement: Additional file 3 — Summary of the Mass Spectrometry results for all 10 samples. [file ar2637-S3.doc]

**Additional file #3.** Summary of the Mass Spectrometry results for all 10 samples

| **Patient ID #** | **All identified proteins** | **Proteins with**  **95% conf.** | **PP spectra matching criteria** |
| --- | --- | --- | --- |
|  |  |  |  |
| 10611 | 674 | 338 | 64,074 |
| 10612 | 531 | 255 | 49,114 |
| 10613 | 885 | 428 | 68,588 |
| 10616 | 753 | 339 | 74,095 |
| 10618 | 1033 | 533 | 70,664 |
| 10619 | 675 | 386 | 64,982 |
| 10620 | 652 | 335 | 88,074 |
| 10621 | 476 | 327 | 100,573 |
| 10622 | 511 | 327 | 93,336 |
| 10623 | 777 | 462 | 91,871 |
|  |  |  |  |
