# Supplementary material for: The effects of infliximab therapy on the serum proteome of rheumatoid arthritis patients
Source: Arthritis Res Ther. 2009 Mar 6;11(2):R32. doi: 10.1186/ar2637 (PMC2688177; doi:10.1186/ar2637)
Supplement: Additional file 4 — Summary of the numbers of proteins observed displaying alterations in expression levels following treatment (# of proteins P ≤ 0.001–0.05, EF ≤ 2.0, ≥ 95% confidence for identification). [file ar2637-S4.doc]

**Additional file #4.** Summary of the numbers of proteins observed displaying alterations in expression levels following treatment (# of proteins p ≤ 0.001–0.05, EF ≥ 2.0, ≥ 95% confidence for identification)

| **Patient ID #** | **# of proteins up-regulated** | **# of proteins down-regulated** |
| --- | --- | --- |
|  |  |  |
| 10611 | 35 | 35 |
| 10612 | 28 | 34 |
| 10613 | 38 | 51 |
| 10616 | 29 | 44 |
| 10618 | 47 | 50 |
| 10619 | 29 | 53 |
| 10620 | 36 | 59 |
| 10621 | 38 | 55 |
| 10622 | 55 | 52 |
| 10623 | 28 | 52 |
|  |  |  |
