# Supplementary material for: The effects of infliximab therapy on the serum proteome of rheumatoid arthritis patients
Source: Arthritis Res Ther. 2009 Mar 6;11(2):R32. doi: 10.1186/ar2637 (PMC2688177; doi:10.1186/ar2637)
Supplement: Additional file 5 — Proteins present in any of the R group that displayed ≥2 fold changes in relative expression levels. (P ≤ 0.001–0.05, EF ≤ 2.0, ≥ 95% confidence for identification). [file ar2637-S5.doc]

**Additional file #5.** Proteins present in **any** of the R group that displayed ≥ 2 fold changes in relative expression levels. (p ≤ 0.001–0.05, EF ≥ 2.0, ≥ 95% confidence for identification)

|  |  |  |
| --- | --- | --- |
| **Name of the protein** | **Ratio (T12/T0)** | **Protein ID (gi #)** |
|  |  |  |
| Serpin peptidase inhibitor, clade A | 0.38 | gi|34785976 |
| Pregnancy zone protein | 0.44 | gi|35825 |
| Fetuin-like protein IRL685 | 0.45 | gi|6562434 |
| C-reactive protein, pentraxin-related | 0.46 | gi|55665343 |
| Angiotensinogen (serine (or cysteine) | 0.47 | gi|37790798 |
| Alpha2-HS glycoprotein | 0.35 | gi|2521981 |
| OTTHUMP00000028705 | 0.46 | gi|56417657 |
| C-reactive protein | 0.35 | gi|30224 |
| Orosomucoid 1 | 0.37 | gi|55958974 |
| Mutant coagulation factor IX | 0.41 | gi|30841428 |
| Mutant beta-globin | 0.41 | gi|18418633 |
| Inter-alpha (globulin) inhibitor H4 | 0.43 | gi|31542984 |
| Serum albumin precursor | 0.50 | gi|6013427 |
| Hemoglobin alpha-1 globin chain | 0.32 | gi|13650074 |
| Serum amyloid A1 isoform 2 | 0.35 | gi|40316910 |
| Truncated coagulation factor IX | 0.44 | gi|30841430 |
| Antithrombin III variant | 0.44 | gi|576554 |
| Hypothetical protein | 0.43 | gi|34365168 |
| Unnamed protein product | 2.46 | gi|34535866 |
| Immunoglobulin heavy chain variable region | 2.02 | gi|3832687 |
| Immunoglobulin kappa chain V-J region | 2.07 | gi|1235771 |
| Immunoglobulin kappa light chain VLJ region | 2.38 | gi|21669339 |
| Unnamed protein product | 2.91 | gi|1335160 |
| Complement factor B | 4.09 | gi|14124934 |
| Thymosin-like 4 | 3.22 | gi|55958822 |
| Keratin 10 | 2.12 | gi|40354192 |
| Serine (or cysteine) proteinase inhibitor, clade A | 2.16 | gi|50363219 |
| Unnamed protein product | 4.98 | gi|34526220 |
| Beta globin chain variant | 2.40 | gi|26892090 |
| Immunoglobulin kappa light chain | 2.68 | gi|33235618 |
| Immunoglobulin heavy chain | 3.70 | gi|46254327 |
|  |  |  |
