# Supplementary material for: The effects of infliximab therapy on the serum proteome of rheumatoid arthritis patients
Source: Arthritis Res Ther. 2009 Mar 6;11(2):R32. doi: 10.1186/ar2637 (PMC2688177; doi:10.1186/ar2637)
Supplement: Additional file 6 — Proteins present in any of the NR group that displayed ≥ 2 fold changes in relative expression levels. (P ≤ 0.001–0.05, EF ≤ 2.0, ≥ 95% confidence for identification). [file ar2637-S6.doc]

**Additional file #6.** Proteins present in **any** of the NR group that displayed ≥ 2 fold changes in relative expression levels. (p ≤ 0.001–0.05, EF ≥ 2.0, ≥ 95% confidence for identification)

| **Name of the protein** | **Ratio (T12/T0)** | **Protein ID (gi #)** |
| --- | --- | --- |
|  |  |  |
| hp2-alpha | 0.25 | gi|296653 |
| Haptoglobin | 0.26 | gi|1620396 |
| A4 amyloid protein precursor | 0.26 | gi|871360 |
| Hemoglobin alpha-1 globin chain | 0.31 | gi|13650074 |
| Osteonectin | 0.33 | gi|338325 |
| Proteoglycan 1, secretory granule | 0.34 | gi|55665173 |
| Mutant beta-globin | 0.36 | gi|18418633 |
| KIAA1106 protein | 0.37 | gi|14133237 |
| Precursor polypeptide (AA -31 to 1139) | 0.40 | gi|37465 |
| Apolipoprotein F precursor (Apo-F) | 0.41 | gi|2492916 |
| Thrombospondin 1 precursor | 0.42 | gi|40317626 |
| Immunoglobulin heavy chain | 0.46 | gi|29837021 |
| Keratin 10 | 0.50 | gi|40354192 |
| Immunoglobulin J polypeptide | 2.05 | gi|24660378 |
| Immunoglobulin kappa light chain VLJ region | 2.10 | gi|21669347 |
| Unnamed protein product | 2.52 | gi|37202 |
| Thymosin-like 4 | 4.46 | gi|55958822 |
|  |  |  |
